# Supplementary material for: Biophysical Properties of Somatic Cancer Mutations in the S4 Transmembrane Segment of the Human Voltage-Gated Proton Channel hHV1
Source: Biomolecules. 2025 Jan 21;15(2):156. doi: 10.3390/biom15020156 (PMC11853527; doi:10.3390/biom15020156)
Supplement: Supplementary file 1 [file biomolecules-15-00156-s001.zip › biomolecules-3379830-supplementary.pdf]

Supplementary Materials

Biophysical Properties of Somatic Cancer Mutations in the S4 Transmembrane Segment of the Human Voltage-Gated Proton Channel hH_V1

1. Detection of HCN1 somatic mutations in cancer according to available databases.

Table S1. H_V1 cancer mutations found in COSMIC, TCGA and ICGC databases.

A2T (C)
E6K (CI)
T10I (CTI)
R11C (TI)
R12S (C)
E19D (CI)
R20M (CI)
R20S (I)
M21I (C)
S22R (CTI)
F24L (CTI)
L25* (CI)
L25V (I)
T29T (CI)
T29M (CT)
V30V (CI)
V31M (C)
G32E (C)
D33D (C)
D34E (I)
H36D (CTI)
A37T (I)
W38* (CI)
W38C (C)
I40S (C)
I40I (CTI)
N41N (CTI)
Y42C (CI)
Y42S (C)
K43N (TI)
W45R (C)
W45L (C)
E48D (CI)
E49E (C)
E50* (CI)
E51D (CI)
E51K (CTI)
E51E (CTI)
E53K (CTI)
E53E (CTI)
V54I (I)
E55K (CTI)
E55Q (T)
Q56E (CI)
P57T (I)
P58S (C)
P59P (CTI)
P61Q (C)
S63S (C)
G64G (CTI)
G64D (C)
E65* (CI)
E65K (T)
E66K (CTI)
A69V (CTI)
A69P (T)
A70V (CI)
A70E (I)
P72P (CTI)
D73D (CTI)
D73G (T)
V74I (CTI)
P80P (CI)
A81T (I)
R83M (CI)
R89S (C)
R89M (CI)
M91I (C)
M91T (C)
L92L (CTI)
L92F (T)
R93R (CTI)
F96F (CTI)
S97R (CI)
R100K (C)
Q102* (CI)
Q102R (C)
V103V (CT)
I104T (I)
D112G (CTI)
A113V (C)
L117F (CTI)
L124P (C)
L124L (CTI)
K125M (C)
I126I (CTI)
P129P (CI)
N132* (C)
A136A (I)
H140Y (CI)
S143S (CTI)
S143R (I)
L147F (CI)
V148V (TI)
F150C (CTI)
M152I (C)
K157N (CTI)
V160L (CTI)
R162C (C)
R162H (I)
H168R (CTI)
F170S (C)
E171Q (CI)
I172T (C)
I172I (T)
A175V (C)
V176I (CI)
V176V (CTI)
V177M (CTI)
V180F (C)
S181* (T)
L184H (CTI)
L184L (CTI)
I186I (T)
L189M (I)
F190F (CTI)
Q191H (I)
Q194K (CI)
E196Q (CTI)
A197V (I)
G199C (CTI)
L201L (CTI)
L203M (C)
L204I (TI)
R205W (CI)
R205R (C)
R208W (CTI)
R208Q (CTI)
V209L (C)
R211R (CI)
I212S (TI)
I212I (I)
G215E (C)
G215R (CI)
I217I (CTI)
V220G (CTI)
R223C (CTI)
R223S (T)
R226Q (CTI)
R226W (CTI)
R230K (CTI)
R230R (CTI)
L231* (C)
L231F (C)
I236M (I)
Q237K (CTI)
Q237* (T)
L238L (TI)
L238F (C)
A239A (CTI)
A240T (CTI)
Q243* (CI)
E246Q (CTI)
Q254K (I)
I256M (TI)
L262L (I)
R264Q (C)
R264R (C)
R264* (TI)
L269L (T)
G270D (CTI)

databases:
C: COSMIC
T: TCGA
I: ICGC

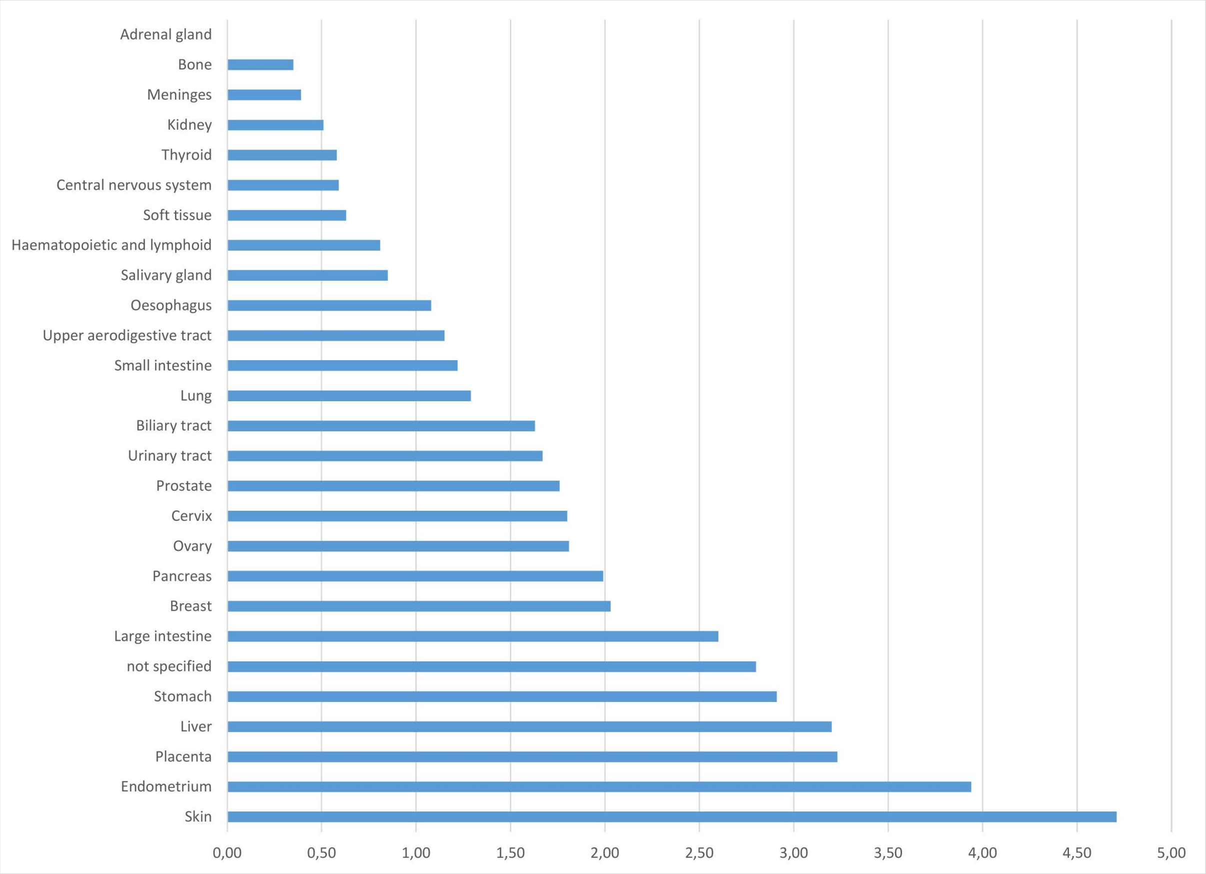

Figure S1. Distribution of the somatic H_V1 mutations within different tumor types according to the COSMIC database.

Table S2. H_V1 structural "hot spots" for somatic mutations.

Hv1 Region	Amino Acid No.	Residues with Mutations	Total Mutation/Residue
N-terminal 1	1-20	6/20 30.0%	0.40
N-terminal 2	21-75	40/55 72.7%	1.07
N-terminal 3	76-100	10/25 40.0%	0.64
S1-S3	101-189	34/89 38.2%	0.53
S4	190-219	16/30 53.3%	0.87
C-terminal 1	220-240	10/21 47.6%	1.05
C-terminal 2	240-273	9/33 27.3%	0.36

Table S3. Mutations in the S4 transmembrane alpha-helix of the human voltage-gated proton channel H_V1.

Mutation	Patient	Tissue	Histology	Somatic Status	LOH	SIFT	Polyphen	Frequency
R205W<br>ENST00000548312.12:c.613C>T	61y,m	skin	malignant melanoma	confirmed	unknown	0.03<br>del	1.000<br>pr.dam.	0.03%
R208W<br>ENST00000548312.12:c.622C>T	46y,m	large intestine	adenocarcinoma	confirmed	unknown	0<br>del	1.000<br>pr.dam.	0.07%
R208W	77y,f	large intestine, caecum	adenocarcinoma	confirmed	unknown			
R208Q<br>ENST00000548312.12:c.623G>A	81y,f	endometrium	endometrioid carcinoma	confirmed	unknown	0.01<br>del	0.998<br>pr.dam.	0.14%
R208Q	unknown	large intestine	adenocarcinoma	confirmed	unknown			
R208Q	82y,f	lung	adenocarcinoma	previously reported[1]	unknown			
R208Q	unknown	heamatopoetic & lymphoid	lymphoid neoplasm	confirmed	unknown			
G215R<br>ENST00000548312.12:c.643G>A	71y,m	lung	squamous cell carcinoma	confirmed	unknown	0<br>del	0.986<br>pr.dam.	0.03%
G215E<br>ENST00000548312.12:c.644G>A	71y,m	skin	squamous cell carcinoma	confirmed	unknown	0<br>del	0.981<br>pr.dam.	0.03%

LOH: Loss of Heterozygosity

1 - Imielinski et al., Journal Cell, 2012;150(6):1107-20; del - deleterious; pr.dam. - probably damaging

2. Patch clamp whole-cell records

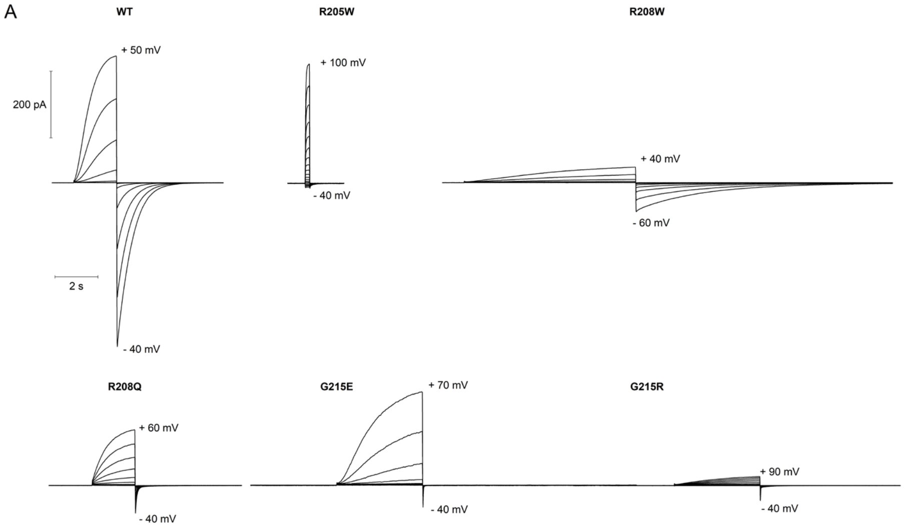

Figure S2. **Proton currents shown in Figure 2A displayed with the same current and time scales.**

3. Structural bioinformatics

Amino acid substitution at the sequence level may lead to subtle structural variation but pronounced functional differences in the mutant structures as compared to the wild-type (WT) structure. Therefore, we explored the dynamics of the mutant channels using molecular dynamics simulations and compared to the WT channel to address the effects of mutations on the structure and possibly on the function.

The voltage-gated proton channel probably has more than one single closed and one single open states [Villalba-Galea 2014, Lee 2018, Cherny 1995]. Here, we used one structure representing the channel in a state that we expect to be impermeable to protons (not conducting) and one structure that should allow the conduction of protons across the membrane. We will distinguish the two structures by referring to them as the “deactivated” (not able to conduct) and “activated” (able to conduct) configurations. An essential structural feature that distinguishes the two configurations is the outward displacement of helix S4 during activation. Although there are still controversies about the extent of the displacement, at least the second (R2) of the three (R1-R3) voltage-sensing arginines in S4 [Bezanilla 2000, DeCoursey 2018] passes across the hydrophobic gasket (HG) that separates the inner and outer vestibules of the channel. The HG is formed by four hydrophobic residues: V109 in S1, F150 in S2 and V178 and V179 in S3 [Bahn 2019]. That at least R2 passes the HG is supported by several studies [Jardin 2022, Li 2015, Kulleperuma 2013].

Construction of the structural models. To date, there is no reliable experimental structure for the human voltage-gated proton channel. A structural model was generated with RobeTTAFold [Baek 2021] from the full-length amino acid sequence of the wild-type channel, residues 1 to 273 as deposited in Uniprot access code Q96D96 [Uniprot 2023], and subsequently reduced to the transmembrane domain, from residue M91 to S224. Especially, RobeTTAFold predicted the HG formed by V109, F150, V178 and V179 and the selectivity filter, D112 [Musset 2011], was located correctly directly above it. R2 was predicted slightly below the HG, in the internal vestibule as awaited in a deactivated configuration. None of the predictions with RobeTTAFold placed R2 above the HG in the external vestibule, as it would be expected in an activated configuration. Therefore, a structural model of the activated state was constructed via homology modeling to the up (active) state of Ci-VSP, available in ModelArchive at https://www.modelarchive.org/doi/10.5452/ma-ehsti [Shen 2022], with Modeller v. 10.2 [Šali 1993] following the approach used in previous works [Jardin 2020, Jardin 2022].

The mutations R205W, R208Q, R208W, G215E and G215R were introduced in the structural models in DS Visualizer. In the deactivated state of the R208W and R208W mutants, rotamers were used, from the Ponder&Richards Library, to prevent steric clashes with the HG. Also, a rotamer of G215R was used to better fit the sidechain of the mutated residue into the environment formed by the sidechains of surroundings residues. There was no need to use alternative rotamers when introducing the mutations in the active state model.

Preparation of the systems for the Molecular Dynamics simulations. Each structural model (one WT and 5 mutants, each in deactivated and activated configurations, resulting in 12 channels) was oriented in the membrane with OPM [Lomize 2012] and embedded in a POPC bilayer and in a solution of water and Na^+ and Cl^- ions, to neutralize the charge of the system and get a salt concentration of 150 mL using CharmmGUI [Jo 2008, Wu 2014]. The resulting systems had similar sizes, about 80 x 80 x 87 Å. For each of the systems (protein + lipid + water + ions), the input files for the molecular dynamics (MD) simulations with AMBER were generated by CharmmGUI at the end of the procedure [Brooks 2009, Lee 2016].

Molecular Dynamics simulations. For each system, a molecular dynamics (MD) simulation was run with AMBER20 [Case 2020], using the FF19SB force field (FF) for protein [Tian 2019], the Lipid21 FF for lipid [Dickson 2022], the TIP3P model for water [Jorgensen 1983] and the Cheatham-Joung parameters for the ions [Joung 2008]. The systems were minimized, heated and equilibrated using the protocol provided automatically by CharmmGUI. This protocol was slightly modified in order to keep small constraints (1.00 kcal.mol.$Å^{-2}$) on the protein backbone until the end of the equilibration (total equilibration time: 1.875 ns per system) and during the first 300 ns of the production of each system, in order to allow the membrane to equilibrate before removing the constraints on the protein. After release of the remaining constraints, each system was simulated for further 5 µs, resulting in simulation time of 5.301,875 ns per system, and a cumulative simulation time for all the systems of 63.622,500 ns. The atomic coordinates were saved every 100 ps for each system, i.e. one snapshot every 100 ps along the trajectory. Later, we will alternatively use the terms frame or structure for a snapshot collected along the trajectory of a channel. The trajectories were analyzed with the CPPTRAJ module of AMBER and with with modules of VMD [Aksimentiev 2005], as specified in the text or in SI. Graphics were generated either with xmgrace or with gnuplot.

Trajectory analysis

Stability assessment. To assess the overall stability of the WT and mutant channels, we calculated the protein backbone Root Mean Square Deviation (RMSD) from the initial structure and Root Mean Square Fluctuations (RMSFs) with respect to the average positions along the 0.5 µs of the unconstrained trajectories. The RMSD is a numerical measurement representing the difference between two structures, a target structure and a reference. The RMSD is typically plotted vs. time and indicates how a structure change over time as compared to the reference. Since the starting structure is the same for all the channels, WT and mutants, in the deactivated and in the activated configuration respectively, we considered the starting structure as the reference. The RMSD can be used to identify large changes in protein structure as compared to the starting point. A leveling off or flattening of the RMSD curve can also indicate that the protein has equilibrated. The RMSD is calculated as:

$$RMSD = \sqrt{\frac{\sum_{i=1}^{n}[m_i(X_i - Y_i)^2]}{M}},$$

where the sum runs over the n atoms considered in the calculation. X_i and Y_i are the coordinate vectors of the target and reference atoms respectively, m_i is the mass of atom i and M is the total mass.

Although the RMSD is indicative of how much the structure deviates from the reference it does not give information about what parts of the protein contribute to the small or large changes during the simulation. For this, we use the RMSF. The RMSF is a measure of the average deviation of atomic positions from their mean positions over time and provides information about the flexibility and dynamics of a structure. With the RMSF, we can assess how much a particular residue moves (fluctuates) during a simulation and thus the individual residue flexibility. Regions with high RMSF values are typically more flexible, while regions with low RMSF values are typically more rigid. RMSF per residue is typically plotted vs. residue number, and can indicate structurally which amino acids in a protein contribute the most to a molecular motion. The RMSF of one atom *i* is calculated as:

$$RMSF_i = \sqrt{\langle (x_i - \langle x_i \rangle)^2 \rangle},$$

where *x* denotes atomic positions and averages are over all input frames.

For the calculation of the RMSDs and RMSFs, one out of every 50 snapshots (every 5 ns) was collected along the trajectory of each channel, resulting in 1000 snapshots per channel and configuration. But the probability distributions of the RMSDs shown in panels C and D of Figure S1$_{MD}$ were calculated using the snapshots generated during the last 2 µs of each MD simulation. For the backbone, we considered the Ca, C N and O atoms. The results are shown in Figures S1$_{MD}$ (RMSD) and S2$_{MD}$ (RMSF). In Figure S2$_{MD}$, the regions for the transmembrane helices are highlighted in red for S1, yellow for S2, green for S3 and blue for S4. For consistency, the regions shown are thus for which a helical secondary structure was found in common in the simulations of all the channels and both in the deactivated and activated configurations: residues 12 to 36 for S1, residues 46 to 72 for S2, residues 81 to 100 for S3 and residues 110 to 132 for S4

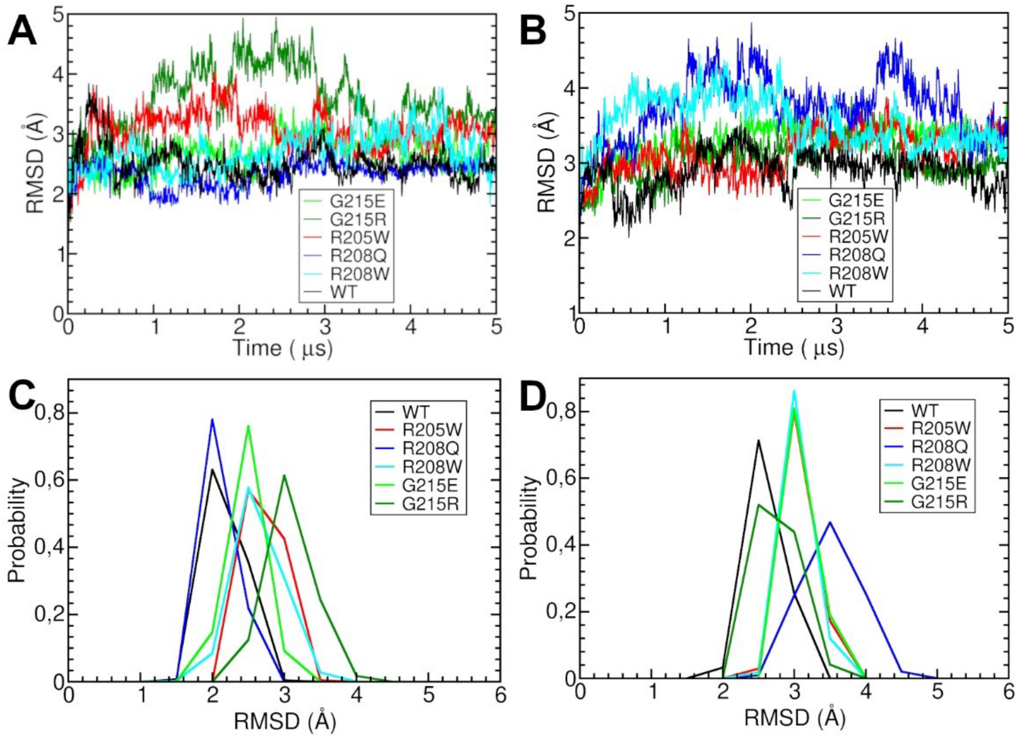

Figure S3. RMSD of the hH$_V$1 channel backbones. (**A**,**B**) Time evolution of the RMSDs of the channel backbones in the deactivated (left) and activated (right) configurations. (**C**,**D**) Probability distribution of the RMSDs during the last 2 µs of the simulations.

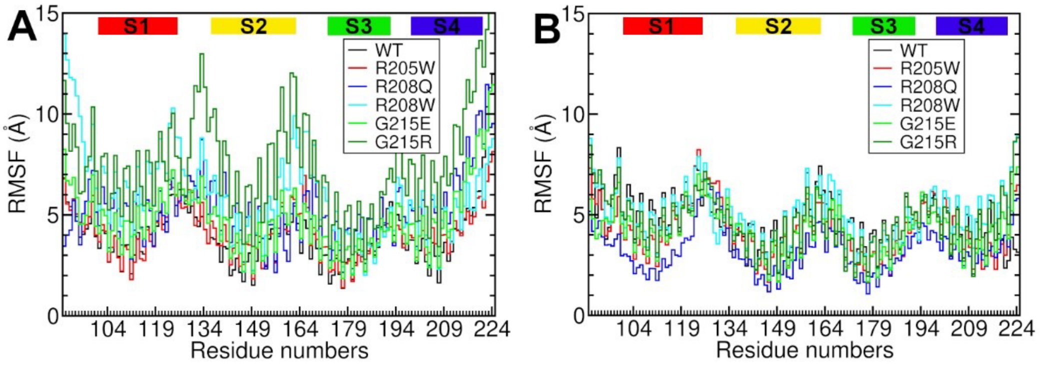

Figure S4. RMSFs of the channel backbones. RMSFs of the channels in the deactivated (**A**) and activated (**B**) configurations. The regions corresponding to the transmembrane alpha helices S1 (red), S2 (yellow), S3 (green) and S4 (blue) are marked on the top.

Clustering. To differentiate the conformational states sampled by the channels during the simulation, the snapshots collected along the trajectory were grouped into clusters of similar protein backbone, based on the Ca, C, N and O atoms of the main chain, for each channel. We used the DBSCAN algorithm [Ester 1996] implemented in cpptraj considering one out of every 10 snapshots (every 1 ns; 5000 snapshots). Previous to clustering with dbscan, K-dist plots were generated with cpptraj to get a rough idea of how to set the dbscan parameters *mindist* and *epsilon*. These parameters differed individually for each clustering (i.e. for each channel and each configuration) and are not reported here. The results of the clustering are summarized in Table S1MD and in Figure S3MD. In ccptraj the clusters are numbered from 0, for the most populated cluster, to X (where X (>0) is the number -1 of generated clusters), for the less populated cluster. For all the channels, the most populated cluster (c0 in Table S1MD and Fig. S3MD) is the cluster populated in the last part of the trajectory (Fig. S3MD), with the exception of deactivated R205W, for which the cluster populated in the last part of the simulation is the second most populated cluster, c1. These clusters also contain a significant portion of the structures generated during the MD simulation of each channel, with always more than 30% (exception: deactivated G215R) and up to more than 80% of the collected snapshots. Consequently, the analyses other than RMSD and RMSF were done using all the structures belonging to the c0 cluster (c1 for deactivated R205W) of the respective channels.

Table S4. Clustering. Information about the last populated cluster.

	Cluster number	centroid	fraction	Number of frames
**WT deactivated**	**c0**	**4471**	**0.455**	**2276**
**R205W deactivated**	**c1**	**4322**	**0.345**	**1723**
**R208Q deactivated**	**c0**	**4264**	**0.480**	**2400**
**R208W deactivated**	**c0**	**2117**	**0.360**	**3276**
**G215E deactivated**	**c0**	**4257**	**0.378**	**1890**

**G215R deactivated**	**c0**	**4218**	**0.219**	**1095**
**WT activated**	**c0**	**2777**	**0.885**	**4426**
**R205W activated**	**c0**	**3869**	**0.491**	**2455**
**R208Q activated**	**c0**	**3334**	**0.433**	**2163**
**R208W activated**	**c0**	**3957**	**0.555**	**2773**
**G215E activated**	**c0**	**1784**	**0.825**	**4173**
**G215R activated**	**c0**	**1611**	**0.850**	**4250**

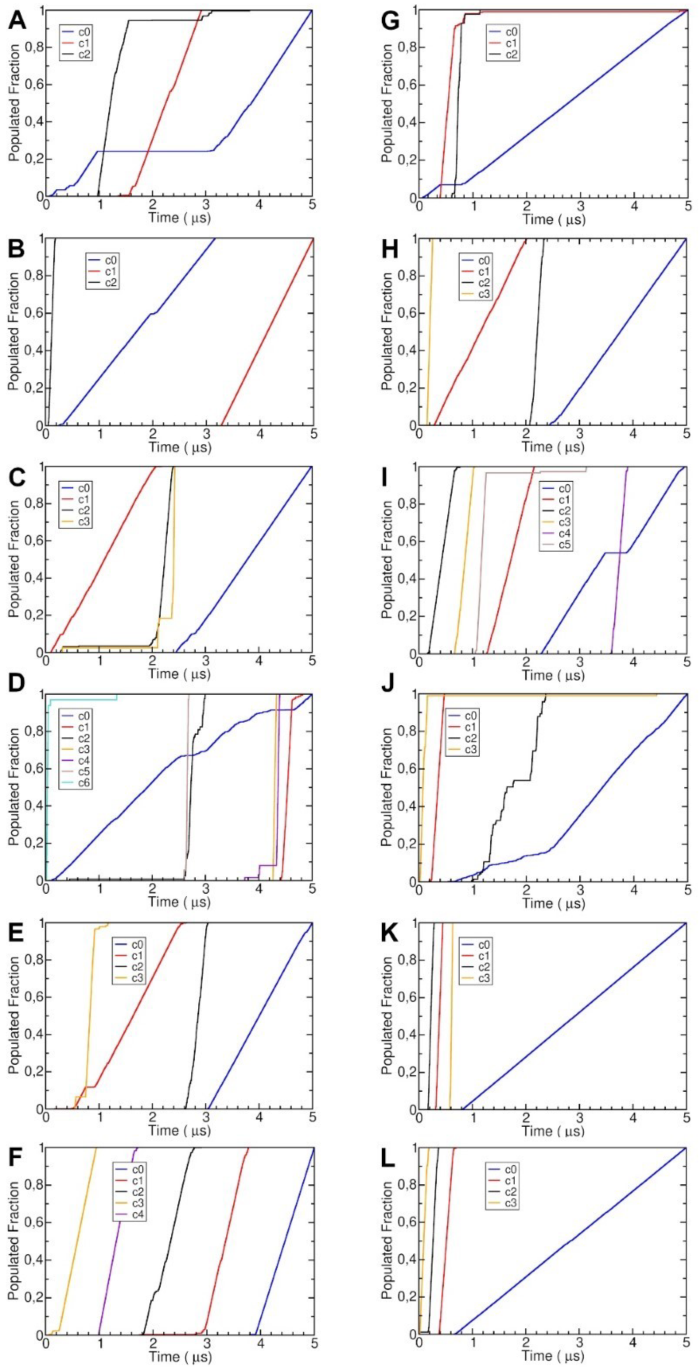

Figure S5. Time evolution of cluster populations. (**A**–**F**) deactivated configurations of WT (**A**), R205W (**B**), R208Q (**C**), R208W (**D**), R208W (**E**), G215E (**F**) and G215R (**G**). (**G**–**L**) activated configuration of WT (**G**), R205W (**H**), R208Q (**I**), R208W (**J**), G215E (**K**) and G215R (**L**). The c0 cluster (blue) is the most and last populated cluster, with exception of deactivated R205W for which c1 (red) is the last but second most populated cluster.

Salt bridged interactions. The distances between the sidechains of the Arg (CZ), Lys (NZ) or His (ND1 and NE2) atoms and Asp (CD) or Glu (CG) were recorded for all the structures of the last populated cluster (c0 but c1 for deactivated R205W) and the probability of the distribution of the distances was calculated with the *pairdist* command of cpptraj by grouping the distances in bins of 0.5 Å. A salt bridge is usually considered to be tight when the distance between the carboxy oxygen atom of an Asp (OD1 or OD2) or Glu (OE1 or OE2) residue and the end sidechain nitrogen atom of an Arg (NH1 or NH2), Lys (NZ) or His (ND or NE) is 4 Å or less [e.g. Kumar 2002]. In order to reduce to one the number of

interactions to be calculated between two residues, e.g. Arg in interaction with Asp or Glu has 4 possible interactions: NH1-OD1, NH1-OD2, NH2-OD1 and NH2-OD2, we considered the CD and CG atoms of Asp and Glu residues respectively, and the CZ atom of Arg residues. In this work, we thus considered to have a tight salt bridge for a distance of 5 Å or less and a probability density greater than 0.5. We also considered weak interactions, when either the distance is less or equal to 5 Å but has a significant probability density, at least 0.2, or when the distance is greater than 5 but smaller than 6 Å. For the R208Q mutation, possible interactions between the glutamine's sidechain amine's nitrogen (NE2) and the carboxy oxygen atom of an Asp or Glu or between the glutamine's sidechain amine's oxygen (OE1) and the sidechain nitrogen atom of an Arg, Lys or His were also considered. The results are shown in Figure S4MD and summarized in Table 1_{MD} and in Figure 1_{MD}.

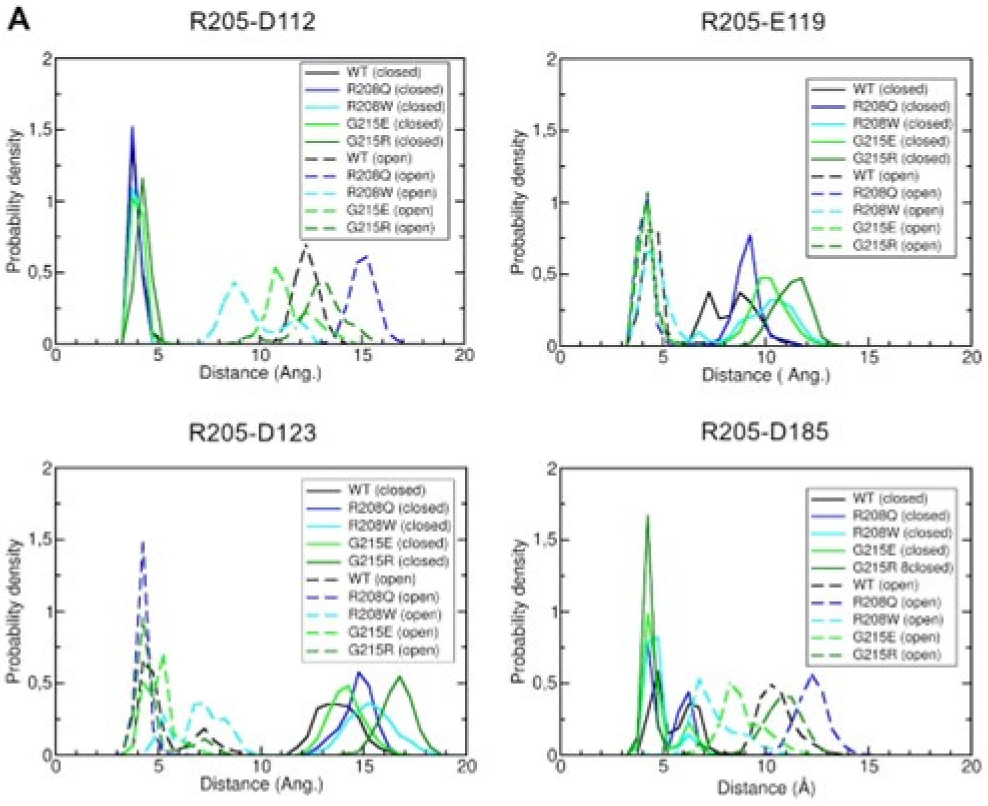

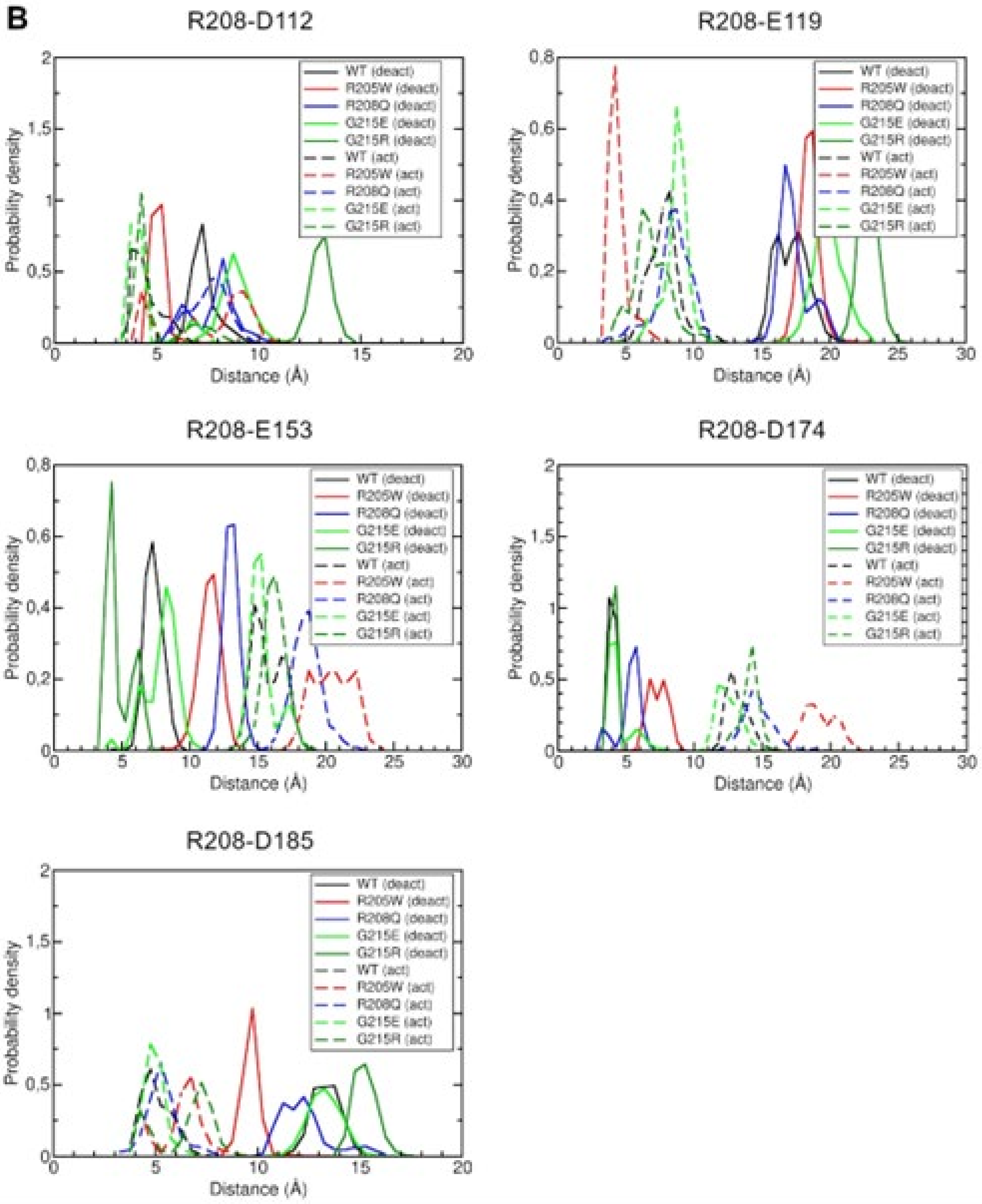
B
R208-D112
R208-E119
R208-E153
R208-D174
R208-D185
Probability density
Distance (Å)
WT (deact)
R205W (deact)
R208Q (deact)
G215E (deact)
G215R (deact)
WT (act)
R205W (act)
R208Q (act)
G215E (act)
G215R (act)

C

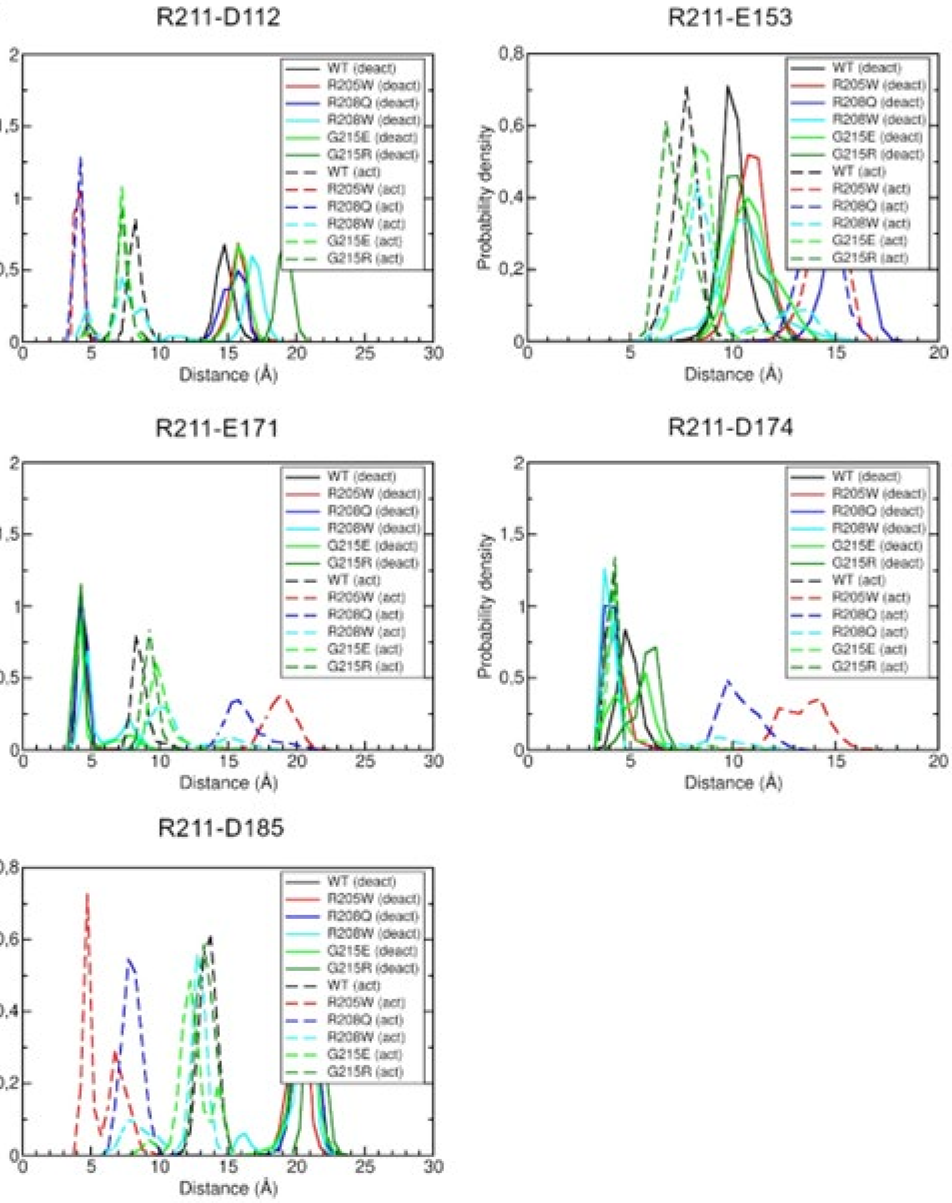
R211-D112
R211-E153
R211-E171
R211-D174
R211-D185
Probability density
Distance (Å)
WT (deact)
R205W (deact)
R208Q (deact)
R208W (deact)
G215E (deact)
G215R (deact)
WT (act)
R205W (act)
R208Q (act)
R208W (act)
G215E (act)
G215R (act)

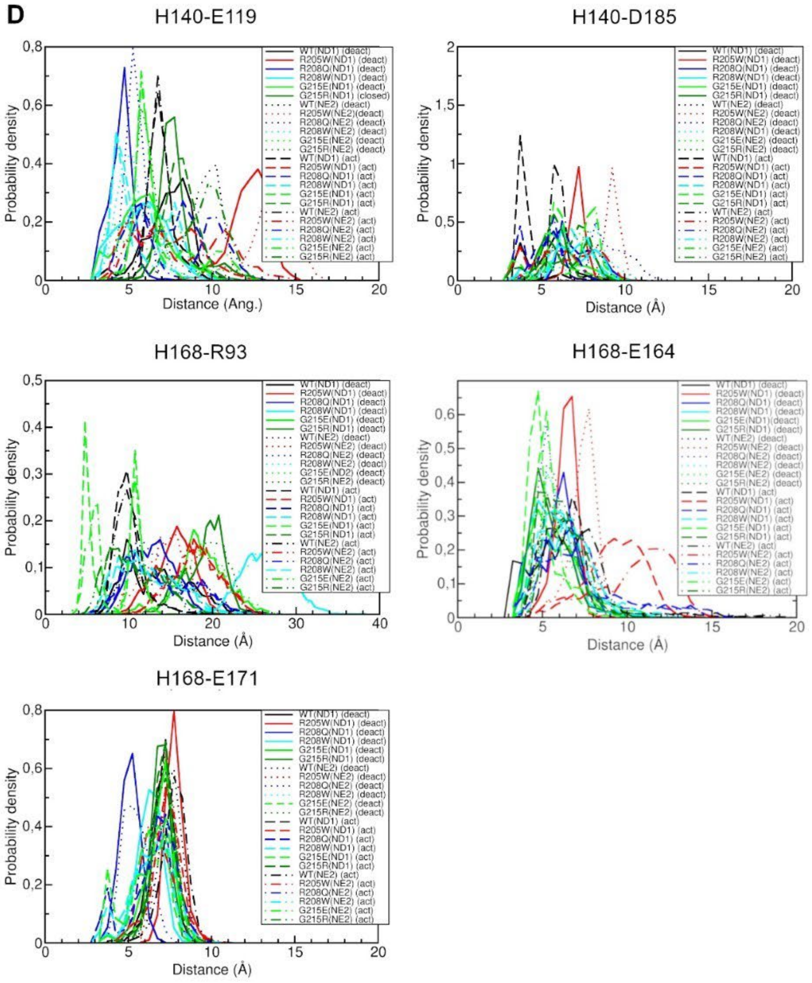
D
H140-E119
Probability density
Distance (Ang.)
H140-D185
Probability density
Distance (Å)
H168-R93
Probability density
Distance (Å)
H168-E164
Probability density
Distance (Å)
H168-E171
Probability density
Distance (Å)

E

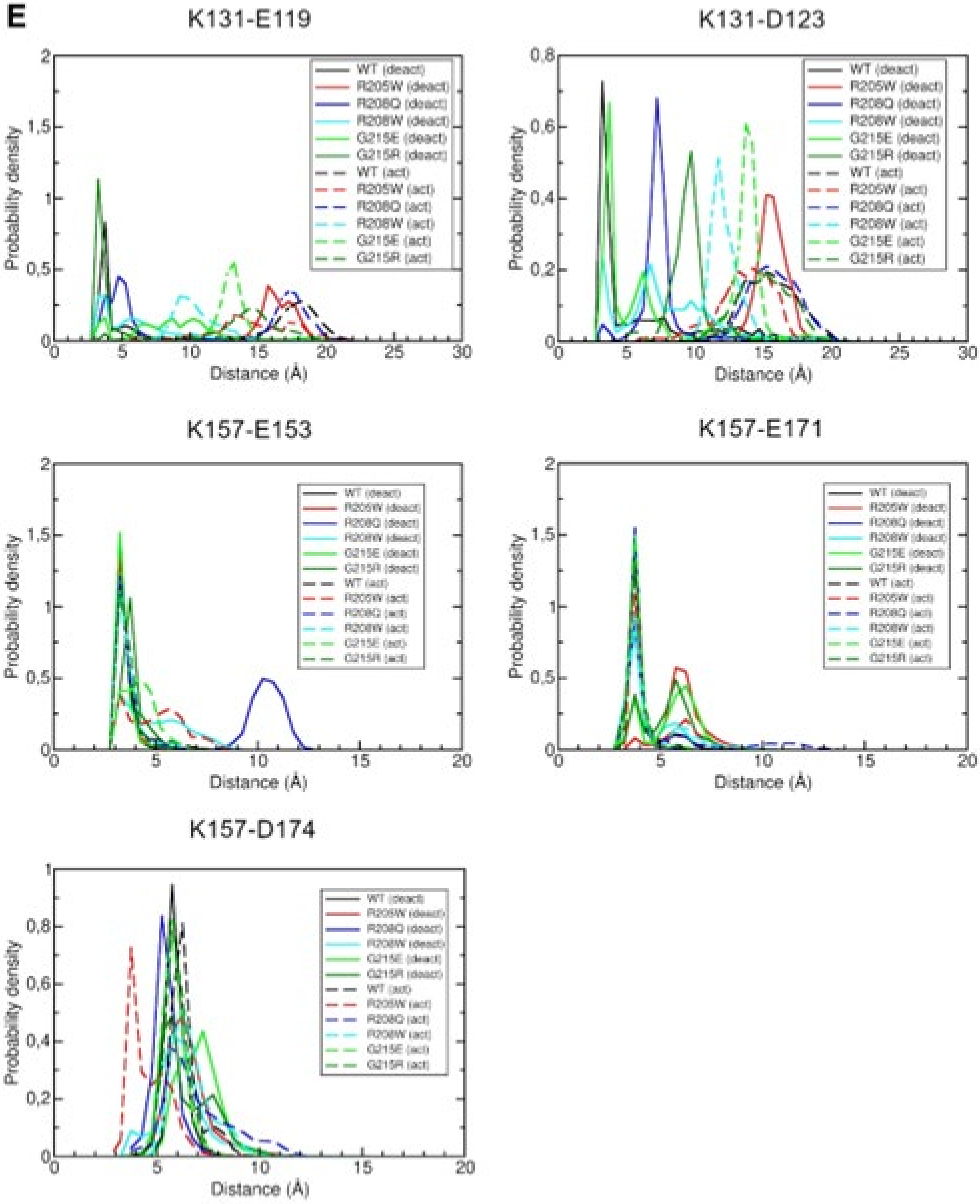
K131-E119
Probability density
Distance (Å)
WT (deact)
R205W (deact)
R208Q (deact)
R208W (deact)
G215E (deact)
G215R (deact)
WT (act)
R205W (act)
R208Q (act)
R208W (act)
G215E (act)
G215R (act)
K131-D123
K157-E153
K157-E171
K157-D174

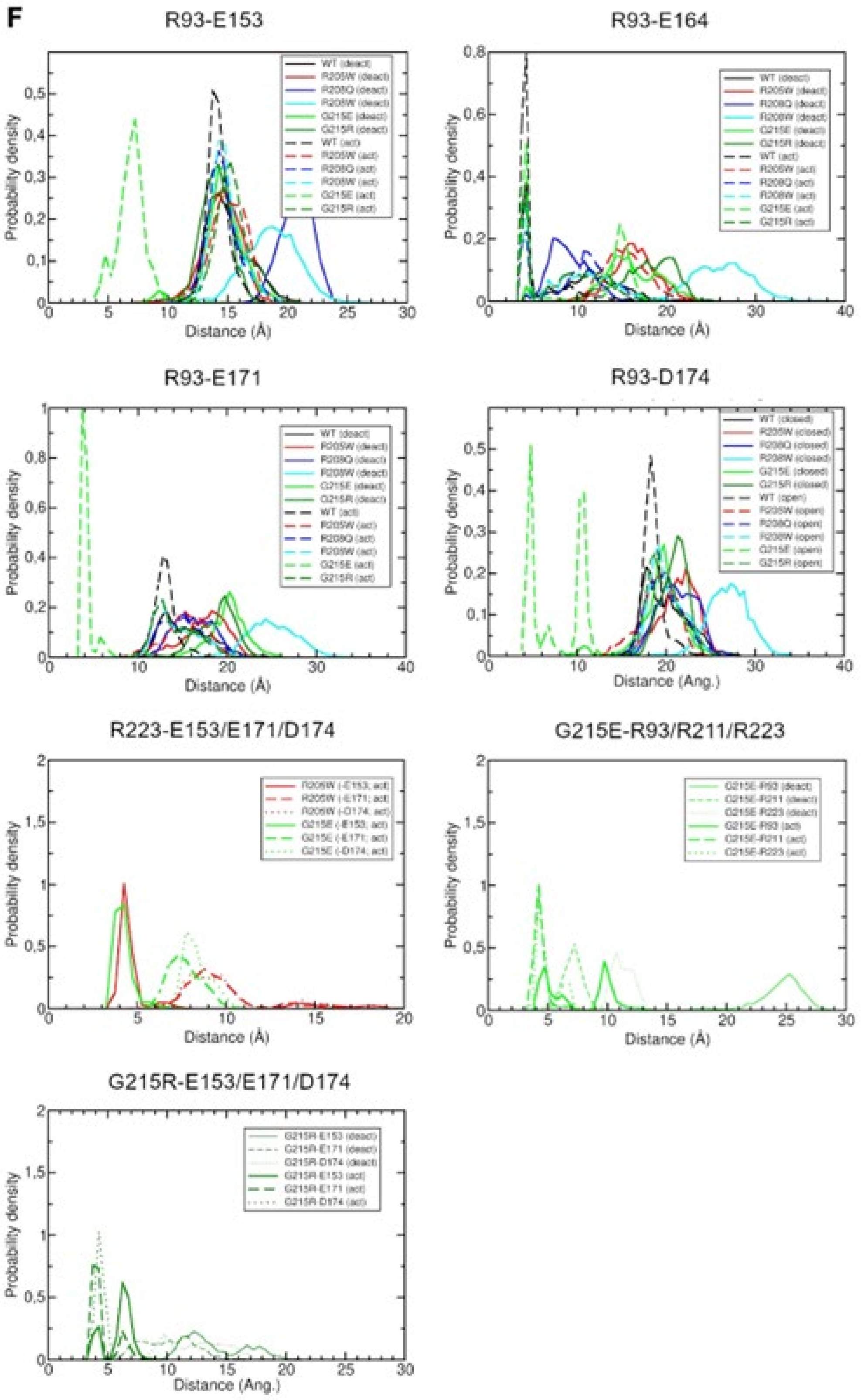

Figure S6. Salt bridges. Probability densities of the salt bridges with the voltage sensing arginines R1 (R205), R2 (R208) and R3 (R211) (**A**–**C**), with the histidines H140 and H168 (**D**), with the lysines K131 and K157 (**E**) and with arginines R93 and R223 and the mutations G215E and G215R in the G215E and G215R channels respectively (**F**).

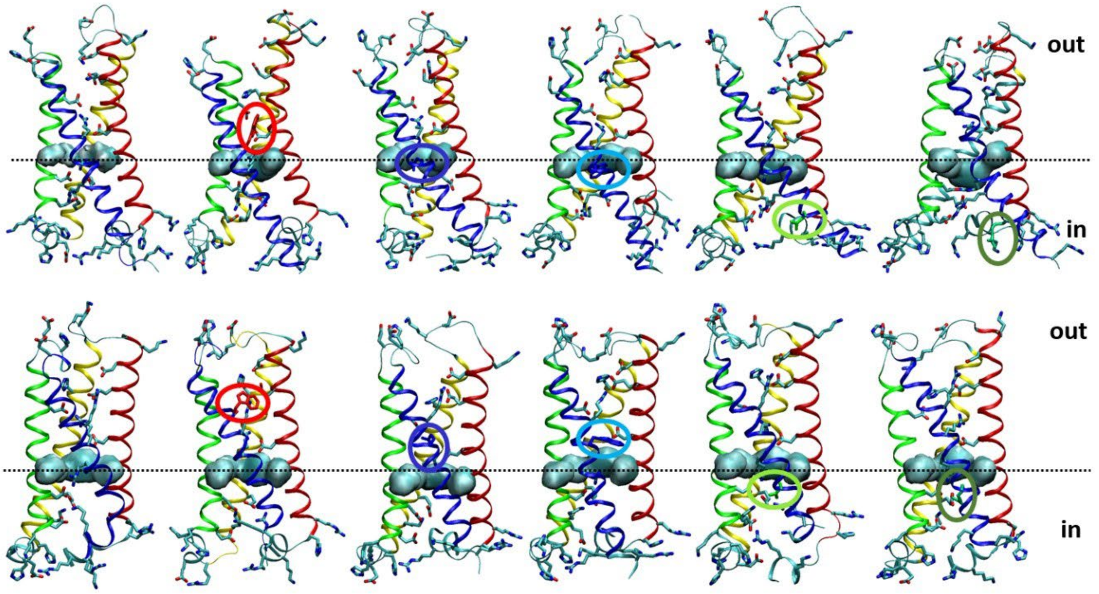

Figure S7. Structure of the cluster centroid. *Top panel*: deactivated channels, *bottom panel*: activated channels. From left to right: WT, R205W, R208Q, R208W, G215E and G215R. Transmembrane helices are shown as ribbons, colored in red for S1, yellow for S2, green for S3 and blue for S4. The mutations are shown as sticks and highlighted with a circle in the corresponding color: red for R205W, dark blue for R208Q, light blue for R208W, light green for G215E and dark green for G215E. The hydrophobic gasket is depicted as a surface. The extracellular vestibule is shown above the HG (out) and the intracellular vestibule below the HG (in).

Radius of gyration. The radius of gyration of the HG was calculated from the backbone Ca atoms of the four hydrophobic residues of the HG (V109, F150, V178 and V179) with and without consideration of the residue of S4 in or close to the HG: R2 in the deactivated configurations and R3 in the activated configurations. The radius of gyration of the HG is generally higher in the activated than in the deactivated configurations, revealing opening of the HG during activation. The results are shown in Figure $S6_{MD}$.

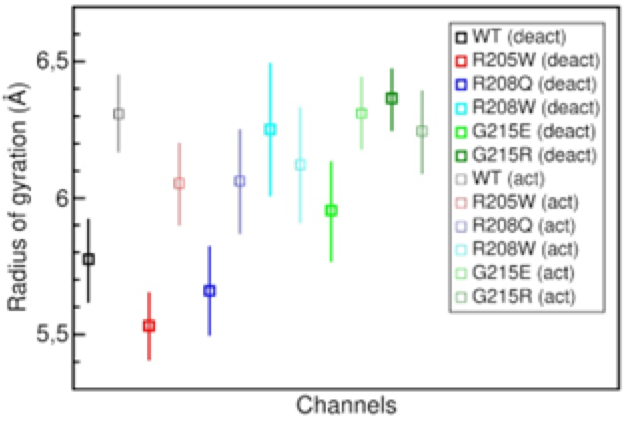

Figure S8. Radius of gyration of the hydrophobic gasket.

R2 shift. The shift of the second voltage sensing arginine, R2 in S4, along the transmembrane axis between the deactivated and activated configurations was calculated from the position of the backbone Ca atom of R2 (CA) or the last heavy atom (CG for R2=R and CZ3 for R2=W) of R2's sidechain

(sc) with respect to the COM of the heavy atoms of the sidechain heavy atoms of the HG residues V109, F150, V177 and V178. This way, the HG COM is at z=0. The results are shown in Figure $S7_{MD}$.

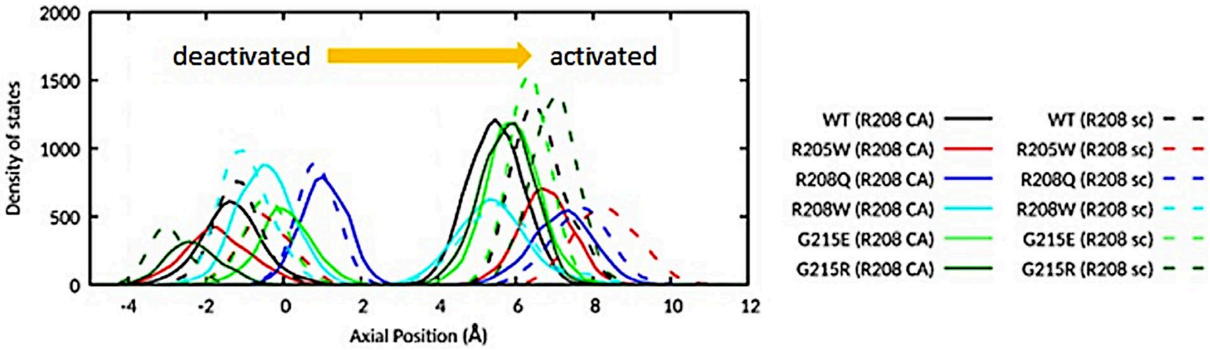

Figure S9. R2 shift. The density of states of the deactivated channels are on the left side of the figure (axial position below 3 Å) whereas those of the activated channels are on the right side (above 3 Å). The densities between the channels and configurations are not directly comparable because the number of structures (from the c0 clusters but c1 for deactivated R205W) used is different for each channel and configuration. Rather, the separation between the density pics in the deactivated and activated configurations reveals the magnitude of R2 shift between the two configurations.

S4 shift. The shift of the S4 helix along the transmembrane axis between the deactivated and activated configurations was calculated from the COM of the backbone heavy atoms (Ca, C, N and O atoms) of the S4 residues with respect to the COM of the heavy atoms of the S1, S2 and S3 residues. Depending on the channel and its configuration, the length of the helices varied slightly in the respective MD simulations. For consistency, we considered in the calculation the residues with a helical secondary structure common in all the channels and configurations: residues 12 to 36 for S1, residues 46 to 72 for S2, residues 81 to 100 for S3 and residues 110 to 132 for S4. The results are shown in Figure S10.

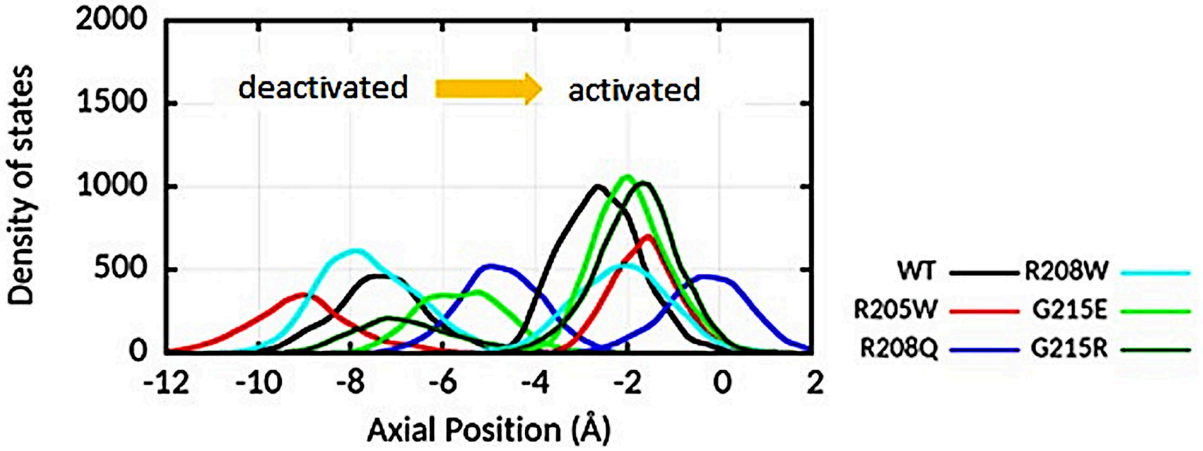

Figure S10. S4 shift. The density of states of deactivated channels are on the left side of the figure (axial position below -4 Å) whereas those of the activated channels are on the right side (above -4 Å). The densities have different heights because the number of structures used is different for each channel and configuration, depending on the number of structures collected in the c0 clusters but c1 for deactivated R205W. The separation between the density pics in the deactivated and activated configurations is relevant for the magnitude of the S4 shift between the two configurations.

Electrostatic potential map. Protein electrostatic potential maps were calculated with the PMEpot plugin of VMD [Aksimentiev 2005].

For the calculation, all the atoms were considered, and a three-dimensional grid of 80 × 80 × 88 points (to ensure at least one grid point per Å in each direction) and an Ewald factor of 0.25 were used. To determine the mean electrostatic potential of a channel, instantaneous electrostatic potentials were calculated for each structure in the last populated cluster of a channel (c0 but c1 for deactivated R205W) and averaged over all the structures in the cluster. The electrostatic potential is obtained by solving the Poisson equation:

$\nabla 2\emptyset(r)=4\pi\sum pi(r)\nabla 2\emptyset r=4\pi\sum pi(r)$

,

where the sum runs over all atoms, and $\rho_i(r)$ is the charge distribution contributed by atom i at position r approximated by a spherical Gaussian:

$\rho i(r)= qi(\beta\pi--\surd)3e-\beta 2|r-ri\ |2\rho ir= qi(\beta\pi)3e-\beta 2|r-ri\ |2$

,

normalized to give the original charge upon integration. q_i is the total charge of atom i.

The electrostatic potential generated by PMEpot is in units of kT/e. Thus, at T = 310 K, one PMEpot unit of electrostatic potential is equivalent to 27 mV. Results are shown in Figure 2_{MD} and in Figures $S9_{MD}$ below.

The electrostatic potential maps in Figure 2MD shows, for a positive charge (here a proton) the attractive (blue) or repulsive (red) force felt by the fixed charged at various points in space. In the figure below, the isoelectrostatic surfaces depicted as red wireframes shows the surface(s) where a proton would feel an attraction at different potentials from -80 to -270 mV.

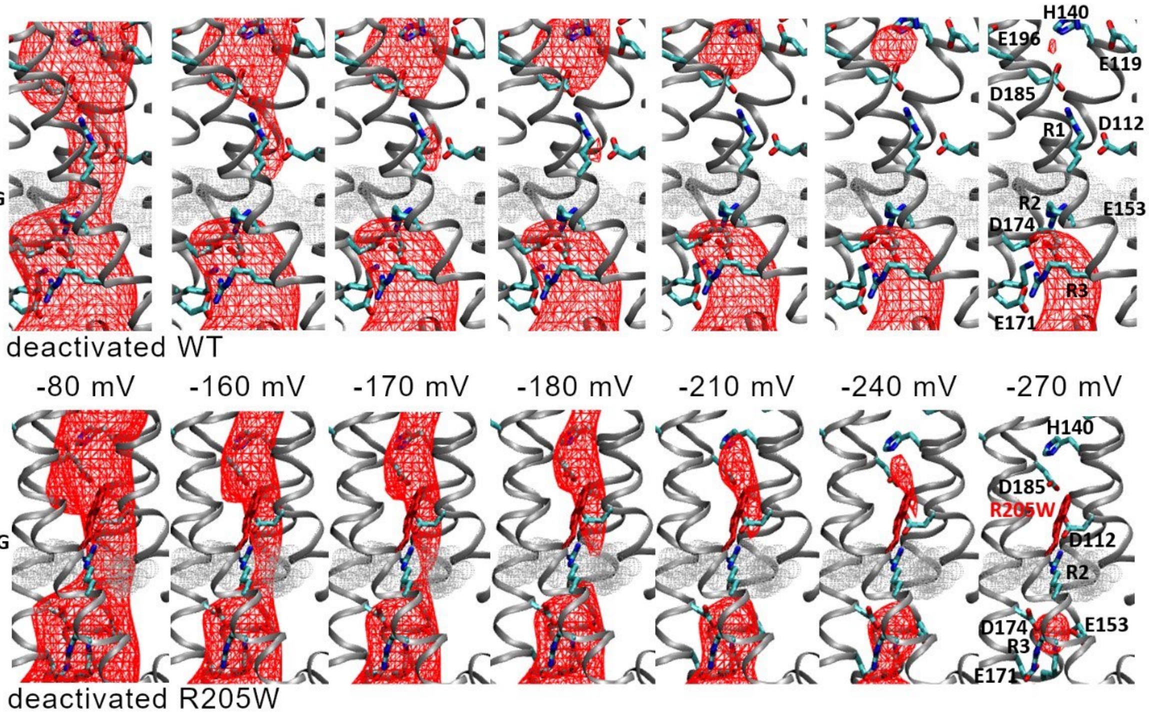

Figure S11. Isoelectrostatic potential. The electrostatic potential isosurfaces of deactivated WT (top) and deactivated R205W (bottom) in the region of the hydrophobic gasket are shown at decreasing

value of the potential, from left to right -80, -160, -170, -180, -210, -240 and -270 mV. For better visibility, the wireframes are not colored according to their isovalue but in red. The potential, that is attractive for a proton, vanishes at higher values in WT than in R205W. Proton hoping between attractive regions in WT might require a potential higher than -0.16 V whereas in R205W it might happen down to -0.21 V.

Water number. Water numbers along the membrane normal in the WT and R205W channels have been calculated for the last populated clusters with the *density* command of cpptraj. For the calculation, the structures have been translated such that Z=0 is at the center of mass of the HG (residues V109, F150, V18 and V179), with the Z-axis being parallel to the membrane normal. Results are shown in Figure $S10_{MD}$.

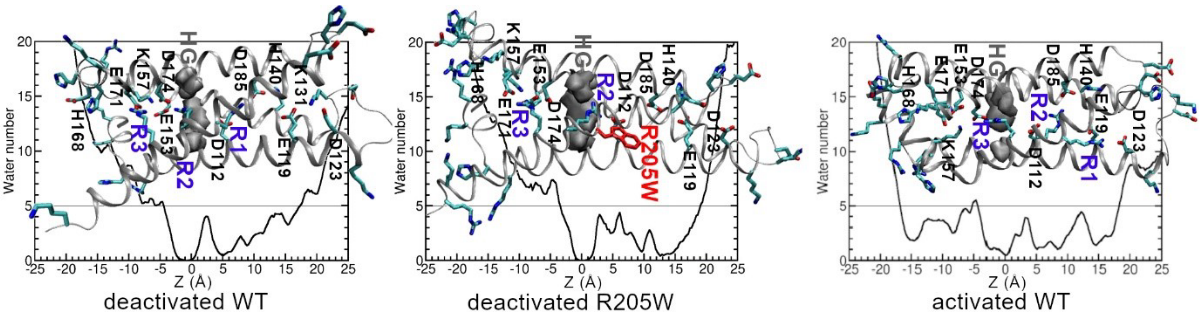

Figure S12. Water profiles. The water profiles were calculated with the *density* command of cpptraj using a cylinder of radius 10 Å along the z-axis and are averages (black lines) over all frames in the last populated cluster. The structure shown is that of the centroid of the last populated cluster. The thin black line corresponding to 5 water molecules only serves the purpose to emphasize the differences between the 3 water profiles, especially between -10 and +10 Å.

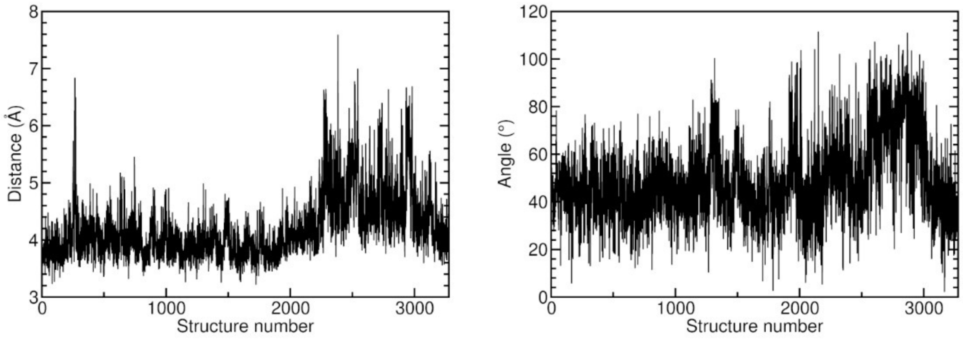

Figure S13. Interaction between R208W and the HG. The distance (left panel) between the center of mass (COM) of the sidechain of the second voltage-sensing arginine, R208, mutated to tryptophan and the COM of the sidechain of the phenylalanine in the HG, F150, and the angle (right panel) between the residues sidechain's rings indicate p–p interactions between the two residues in the deactivated configuration. In the activated configuration, R208W's sidechain interacts with lipids in a manner similar to R205W, as shown in Figure 7.

References

1. Baek, M.; DiMaio, F.; Anishchenko, I.; Dauparas, J.; Ovchinnikov, S.; Lee, G.R.; Wang, J.; Cong, Q.; Kinch, L.N.; Schaeffer, R.D.; et al. Accurate prediction of protein structures and interactions using a 3-track network. *Science* **2021**, *373*, 871–876. https://doi.org/10.1126/science.abj8754.
2. Bahn, R.; Cherny, V.V.; Morgan, D.; Musset, B.; Thomas, S.; Kulleperuma, K.; Smith, S.M.E.; Pomès, R.; DeCoursey, T.E. Hydrophobic gasket mutation produces gating pore currents in closed human voltage-gated proton channels. *Proc. Natl. Acad. Sci. USA* **2019**, *116*, 18951–18961. https://doi.org/10.1073/pnas.1905462116
3. Bezanilla F. The voltage sensor in voltage-dependent ion channels. *Physiol. Rev.* **2000**, *80*, 555–592. https://doi.org/10.1152/physrev.2000.80.2.555
4. Brooks, B.R.; Brooks, C.L., III; MacKerell, A.D.; Nilsson, L., Jr.; Petrella, R.J.; Roux, B.; Won, Y.; Archontis, G.; Bartels, C.; Boresch, S.; et al. CHARMM: The Biomolecular Simulation Program. *J. Comput. Chem.* **2009**, *30*, 1545–1614. https://doi.org/10.1002/jcc.21287
5. Case, D.A.; Belfon, K.; Ben-Shalom, I.Y.; Brozell, S.R.; Cerutti, D.S.; Cheatham, T.E., III; Cruzeiro, V.W.D.; Darden, T.A.; Duke, R.E.; Giambasu, G.; et al. *AMBER 2020: Reference Manual (Covers Amber20 and AmberTools20)*; University of California: San Francisco, CA, USA, 2020.
6. Cherny, V.V.; Markin, V.S.; DeCoursey, T.E. The voltage-activated hydrogen ion conductance in rat alveolar epithelial cells is determined by the pH gradient. *J. Gen. Physiol.* **1995**, *105*, 861–896. https://doi.org/10.1085/jgp.105.6.861.
7. DeCoursey, T.E. Voltage and pH sensing by the voltage-gated proton channel, Hv1. *J. R. Soc. Interface* **2018**, *15*, 20180108. https://doi.org/10.1098/rsif.2018.0108.
8. Dickson, C.J.; Ross CWalker, R.C.; Gould, I.R. Lipid21: complex lipid membrane simulatons with AMBER. *J. Chem. Theory Comput.* **2022**, *18*, 1726–1736. https://doi.org/10.1021/acs.jctc.1c01217
9. Ester, M.; Kriegel, H.; Sander, J.; Xu, X. A density-based algorithm for discovering clusters in large spatial databases with noise. *KDD-96 Proc.* **1996**, *96*, 226–231.
10. Imielinski, M.; Berger, A.H.; Hammerman, P.S.; Hernandez, B.; Pugh, T.J.; Hodis, E.; Cho, J.; Suh, J.; Capelletti, M.; Sivachenko, A.; et al. Mapping the hallmarks of lung adenocarcinoma with massively parallel sequencing. *J. Cell* **2012**, *150*, 1107–1120.
11. Kulleperuma, K.; Smith, S.M.; Morgan, D.; Musset, B.; Holyoake, J.; Chakrabarti, N.; Cherny, V.V.; DeCoursey, T.E.; Pomès, R. Construction and validation of a homology model of the human voltage-gated proton channel hHV1. *J Gen Physiol.* **2013**, *141*, 445–465. https://doi.org/10.1085/jgp.201210856.
12. Jardin, C.; Chaves, G.; Musset, B. Assessing structural determinants of Zn2+ binding to human Hv1 via multiple MD simulations. *Biophys. J.* **2020**, *118*, 1221–1233. https://doi.org/10.1016/j.bpj.2019.12.035.
13. Jardin, C.; Ohlwein, N.; Chaves, G.; Musset, B. The pH-dependent gating of the human voltage-gated proton channel from computational simulations. *Phys. Chem. Chem. Phys.* **2022**, *24*, 9964–9977. https://doi.org/10.1039/d1cp05609c.
14. Jo, S.; Kim, T.; Iyer, V.G.; Im, W. CHARMM-GUI: A Web-based Graphical User Interface for CHARMM. *J. Comput. Chem.* **2008**, *29*, 1859–1865. https://doi.org/10.1002/jcc.20945.
15. Jorgensen, W.L.; Chandrasekhar, J.; Madura, J.D.; Impey, R.W.; Klein, M.L. Comparison of simple potential functions for simulating liquid water. *J. Chem. Phys.* **1983**, *79*, 926–935. https://doi.org/10.1063/1.445869.
16. Joung, I.S.; Cheatham, T.E., III. Determination of alkali and halide monovalent ion parameters for use in explicitly solvated biomolecular simulations. *J. Phys. Chem. B* **2008**, *112*, 9020–9041. https://doi.org/10.1021/jp8001614.
17. Kumar, S.; Nusinov, R. Close-range electrostatic interactions in proteins. *ChemBioChem* **2002**, *3*, 604–617. https://doi.org/10.1002/1439-7633(20020703)3:7<604::AID-CBIC604>3.0.CO;2-X.
18. Lee, J.; Cheng, X.; Swails, J.M.; Yeom, M.S.; Eastman, P.K.; Lemkul, J.A.; Wei, S.; Buckner, J.; Jeong, J.C.; Qi, Y.; et al. CHARMM-GUI Input Generator for NAMD, GROMACS, AMBER, OpenMM, and CHARMM/OpenMM Simulations using the CHARMM36 Additive Force Field. *J. Chem. Theory Comput.* **2016**, 12, 405–413. https://doi.org/10.1021/acs.jctc.5b00935.
19. Lee, M.; Bai, C.; Feliks, M.; Alhadeff, R.; Warshel, A. On the control of the proton current in the voltage-gated proton channel Hv1. *Proc. Natl. Acad. Sci. USA* **2018**, *115*, 10321–10326. https://doi.org/10.1073/pnas.1809766115.
20. Li, Q.; Shen, R.; Treger, J.S.; Wanderling, S.S.; Milewski, W.; Siwowska, K.; Bezanilla, F.; Perozo, E. Resting state of the human proton channel dimer in a lipid bilayer. *Proc. Natl. Acad. Sci. USA* **2015**, *112*, E5926–E5935. https://doi.org/10.1073/pnas.1515043112.
21. Lomize, M.A.; Pogozheva, I.D.; Joo, H.; Mosberg, H.I.; Lomize, A.L. OPM database and PPM web server: resources for positioning of proteins in membranes. *Nucleic Acids Res.* **2012**, *40*, D370–D376. https://doi.org/10.1093/nar/gkr703.
22. Musset, B.; Smith, S.M.E.; Rajan, S.; Morgan, D.; Cherny, V.V.; DeCoursey, T. Aspartate D112 is the selectivity filter of the human voltage gated proton channel. *Nature* **2011**, *480*, 273–277. https://doi.org/10.1038/nature10557.

23. Šali, A.; Blundell, T.L. Comparative protein modelling by satisfaction of spatial restraints. *J. Mol. Biol.* **1993**, *234*, 779–815. https://doi.org/10.1006/jmbi.1993.1626.
24. Shen, R.; Meng, Y.; Roux, B.; Perozo, E. Mechanism of voltage gating in the voltage-sensing phosphatase Ci-VSP. *Proc. Natl. Acad. Sci. USA* **2022**, *119*, e2206649119. https://doi.org/10.1073/pnas.2206649119.
25. Tian, C.; Kasavajhala, K.; Belfon, K.A.; Raguette, L.; Huang, H.; Migues, A.N.; Bickel, J.; Wang, Y.; Pincay, J.; Wu, Q.; et al. ff19SB: amino-acid-specific protein backbone parameters trained against quantum mechanics energy surfaces in solution. *J. Chem. Theory Comput.* **2020**, *16*, 528–552. https://doi.org/10.1021/acs.jctc.9b00591.
26. UniProt: the Unversal Protein Knowledgebase in 2023. *Nucleic Acids Res.* **2023**, *51*, D523–D531. https://doi.org/10.1093/nar/gkac1052.
27. Villalba-Galea, C.A. Hv1 proton channel opening is preceded by a voltage-independent transition. *Biophys. J.* **2014**, *107*, 1564–1572. https://doi.org/10.1016/j.bpj.2014.08.017.
28. Wu, E.L.; Cheng, X.; Jo, S.; Rui, H.; Song, K.C.; Dávila-Contreras, E.M.; Qi, Y.; Lee, J.; Monje-Galvan, V.; Venable, R.M.; et al. CHARMM-GUI Membrane Builder Toward Realistic Biological Membrane Simulations. *J. Comput. Chem.* **2014**, *35*, 1997–2004. https://doi.org/10.1002/jcc.23702.
